# Impaired early type I interferon responses to influenza virus infection in aged mice are associated with subsequent increased pulmonary inflammation

**DOI:** 10.1007/s11357-025-01892-3

**Published:** 2025-09-23

**Authors:** Wenxin Wu, Jeremy S. Alexander, J. Leland Booth, Chaoqun Huang, Lin Liu, Craig A. Miller, Douglas A. Drevets, Jordan P. Metcalf

**Affiliations:** 1 Pulmonary, Critical Care & Sleep Medicine, Department of Medicine, University of Oklahoma Health Sciences Center, Room 425, 800 N. Research Pkwy, Oklahoma City, OK 73104 USA; 2 The Lundberg-Kienlen Lung Diseases and Infection Laboratory, Department of Physiological Sciences, Oklahoma State University, Stillwater, OK USA; 3 Department of Veterinary Pathobiology, College of Veterinary Medicine, Oklahoma State University, Stillwater, OK USA; 4 Infectious Diseases, Department of Medicine, University of Oklahoma Health Sciences Center, 800 Stanton L. Young Blvd, Suite 7300, Oklahoma City, OK 73104 USA; 5 Veterans Affairs Medical Center, Oklahoma City, OK USA; 6 Department of Microbiology and Immunology, University of Oklahoma Health Sciences Center, Oklahoma City, OK USA

**Keywords:** Influenza virus, Aging, Interferon, Lung, Inflammation, Innate immunity

## Abstract

Seasonal influenza is responsible for significant mortality and morbidity worldwide. Seventy to ninety percent of these deaths occur in those aged 65 or older. To determine the innate immune responses to influenza A virus (IAV) infection, young (12-week) and old (70-week) C57BL/6 J mice were infected intranasally (i.n.) with IAV PR8. Immune responses were determined by qRT-PCR and single-cell RNA sequencing (scRNA-seq). Old mice, as compared to young mice, had significantly higher viral loads and lower type I interferon (IFN) expression in the lung at 3 days post-infection (dpi). In contrast, at this time point aged mice had significantly higher amounts of type III IFN expression, which correlated with the higher viral loads observed. Histopathology revealed that IAV infection in old mice resulted in lower pathological scores early (at 5 dpi) and higher lung pathological scores of diseases later (at 7 dpi) than in young mice. scRNA-seq analysis revealed that, at 7 dpi, older mice exhibited sustained local inflammatory responses, with higher expression levels of *Ddx58*, *Irf7*, *Il6*, and *Tnf* across various immune cells, including macrophages, monocytes, Natural killer cells, dendritic cells, and granulocytes, compared to young mice. Our murine model of aging and influenza infection demonstrated that aging dysregulated early IFN responses to influenza infection resulting in enhanced viral replication. These altered IFN responses in old mice also result in enhanced lung inflammation late after infection and may increase the incidence of secondary bacterial infections seen in older individuals.

**Graphical Abstract:**

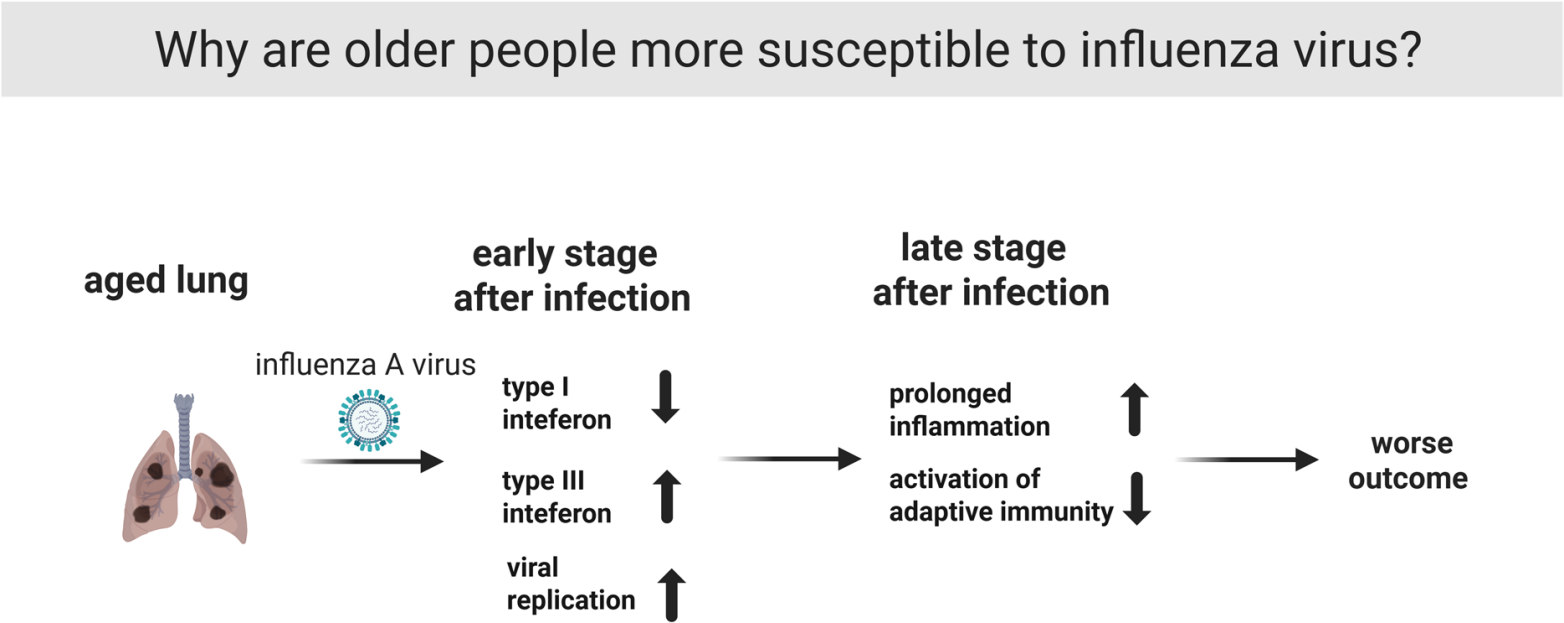

## Background

Respiratory infections caused by seasonal influenza and other viral pathogens are a major cause of hospitalizations globally and are a leading cause of morbidity and especially mortality in the elderly [1]. Specifically, although those older than 70 years of age have similar hospitalization rates from influenza than children less than 5 years of age, mortality from these infections is 35-fold higher in the elderly [2]. This is thought to be due, in part, to older hosts exhibiting delayed immune responses and prolonged inflammation after infections, leading to excessive tissue damage and a greater likelihood of death [3]. Mouse models of influenza infection reveal that aging dysregulates the inflammatory response and increased morbidity and death as also found in patients [4, 5]. Nonetheless, the exact mechanism for enhanced susceptibility to influenza A virus (IAV) infection in the aged is poorly understood.

Interferons (IFNs) and inflammatory cytokines are essential components of innate immune responses to viral infection in the lung [6]. IAV strongly induces IFNs, which mediate host resistance to infection and inhibit viral replication. Host cells, such as airway epithelial cells, macrophages and dendritic cells (DCs) produce IFNs after sensing viral components using several pattern-recognition receptors (PRRs) including Toll-like receptors (TLRs), RIG-I like helicases (RLRs) and nucleotide-binding domain and leucine-rich-repeat-containing proteins (NLRs). IFNs also activate the expression of many IFN-stimulated genes (ISGs) in infected and nearby cells [7]. ISGs have proinflammatory effects and initiate an intracellular antiviral program that prevents the spread of pathogens. IFNs also enhance the production of cytokines and chemokines by innate immune cells, a process that can lead to pathologic tissue damage as well as to beneficial anti-viral host defenses.

IFNs are divided into type I (IFN-α and β), II (IFN-γ) and III (IFN-λ) subtypes. During IAV infection, type I IFNs are primarily produced in alveolar macrophages, plasmacytoid DCs (pDCs) and epithelial cells [8]. Type III IFNs are primarily expressed in epithelial cells and their receptors are mostly distributed in epithelial cells, consistent with their function during mucosal infection. Their primary role is protecting the respiratory tract against viral infection [9, 10]. In mucosa-rich tissues, type III IFNs can largely compensate for a defective type I IFN system [10, 11] and induce less inflammation compared with type I IFNs [12, 13]. However, type III IFNs may also impair lung restoration after viral infection [14, 15] and can increase susceptibility to bacterial infections [16]. Little is known about how aging affects the innate immune system's ability to produce IFNs and activate innate immune cells.

Of critical importance in this context is the fact that seasonal influenza vaccine performs poorly in the elderly. Vaccination only reduces the incidence of hospitalization for influenza and pneumonia by 33% in vaccinees over age 65 [17]. Early innate immune responses, particularly IFN signaling, are crucial for effective control of IAV infection and for the optimal activation of adaptive immunity. Impairment to these initial responses can have significant downstream consequences for the development of robust and long-lasting adaptive immunity [18, 19]. Thus, there is an urgent need to study the effects of aging on the innate immune responses to IAV.

The goal of the work presented here was to determine the degree to which aging increased the risk of lung injury following influenza infection in our murine model and to reveal potential mechanisms underlying this response. Our hypothesis is that early IFN responses to IAV infection are attenuated in the lungs of aged mice, potentially leading to heightened pulmonary inflammation and more severe disease. To achieve this goal, we compared viral loads and innate immune responses in the lungs of young and aged mice during IAV infection.

## Methods

### Influenza A virus and mouse infection

The IAV strain used in this study was A/PR/34/8 (PR8). The stocks were propagated in Madin-Darby canine kidney (MDCK, ATCC, Manassas, VA) cells following standard procedures [20]. The virus was titered by plaque assay in MDCK cells, aliquoted and stored at − 80 °C.

Young (12-week) and old (70-week) C57BL/6 J mice were purchased from Jackson (Bar Harbor, ME). Mice were held in a nose-up, vertical position while sedated and administered by intranasal instillation of virus diluted in PBS (200 PFU/mouse in 70 µl PBS). An equal volume of PBS without virus was inoculated to the mock group. The animals were meticulously watched both during and after each procedure to ensure recovery. Mice were monitored daily for 7 days for clinical symptoms (shaking, inactivity and piloerection) and their weight was recorded daily.

### Measurement of mRNA expression by quantitative real-time PCR (qRT-PCR)

A modified TRIzol (Invitrogen, Carlsbad, CA) procedure was used to extract and quantify the total RNA from the lung. Electrophoresis of agarose gel/formaldehyde was used to confirm the integrity of the RNA. Using the oligo (dT) SuperScript II First-Strand Synthesis System for RT-PCR, equal amounts (1 µg) of RNA from each sample were reverse-transcribed into cDNA (Invitrogen, Carlsbad, CA). Gene specific primers’ sequences are shown in our prior publication [21]. qRT-PCR was carried out on a Bio-Rad CFX96TM Touch Real-Time PCR Detection System using 100 ng sample RNA and SYBR Green (Quanta Biosciences, Gaithersburg, MD). The target gene's ΔCT value and its normalizer, β-actin, were used to calculate and plot the results.

### Histological analysis of mouse lungs

Mice were euthanized on day 5 and 7 after the IAV infection, and the lungs were fixed in 4% paraformaldehyde in PBS for 24 h at room temperature before being embedded in paraffin. Paraffin-embedded sections were trimmed to 5 µm and collected onto charged slides before staining with hematoxylin and eosin (H&E) for evaluation by light microscopy. Lung tissues were evaluated for: alveolar damage, hyaline membrane formation, serous exudate/edema, alveolar fibrin deposition, alveolar histiocytes, perivascular infiltrates, type II pneumocyte hyperplasia, peri-bronchial inflammation, smooth muscle hyperplasia, thrombosis, and fibrinoid vasculitis. All tissues were assigned a quantitative histopathological score based on previously described criteria [22, 23]: 0 = no apparent pathology/change; 1 = minimal change (minimally increased numbers of inflammatory cells); 2 = mild change (mild inflammatory infiltrates, damage/necrosis, fibrin deposition and/or exudation); 3 = moderate change (as previously described, but more moderately extensive); 4 = marked changes (as previously described, but with severe inflammation, damage/necrosis, exudation, vasculitis and/or thrombosis). All tissues were evaluated and scored by a board-certified veterinary pathologist blinded to study groups to eliminate bias and ensure scientific rigor.

### Single-cell RNA sequencing (scRNA-seq)

Seven days after IAV infection, mice were euthanized, and their lungs were perfused. Whole lungs were collected, fixed and frozen according to the Tissue Fixation & Dissociation for Chromium Fixed RNA Profiling kit (10X Genomics, Pleasanton, CA) and the 10 × Genomics Chromium Next GEM Single Cell Fixed RNA Sample Preparation Kit. Dissociation of fixed Tissue and creation of single cell suspension were done according to the manufacturers protocol. Cells were loaded onto the Chromium X (10X Genomics), then the Mouse Transcriptome 10 × Genomics Chromium Fixed RNA Kit, was used to generate cDNA libraries. All samples were handled so that approximately 10,000 cells were captured as 10X Genomics Gel Beads-in-emulsion (GEMs). Barcoded libraries were sequenced on an Illumina NovaSeq X Plus 25B PE300 instrument using the 10X genomics’ suggested cycling conditions. Data were demultiplexed and aligned to the mm10 2020-A reference transcriptome (10 × Genomics) using Cell Ranger (v8.0, 10 × Genomics).

The demultiplexed FASTQ files were analyzed by the Molecular Biology and Genomics Core at the Oklahoma Center for Respiratory and Infectious Diseases using the 'cellranger multi' function, as part of the Cell Ranger v8.0 software suite (10X Genomics). For read mapping, the pre-indexed mouse reference transcriptome (GRCm39 2024-A) was used (available at: https://cf.10xgenomics.com/supp/cell-exp/refdata-gex-GRCm39-2024-A.tar.gz). The analysis employed the appropriate parameters for the Gene Expression Flex Kit (Chromium Next GEM Chip Q Single Cell Kit, 1,000,422) for 16 samples. Additionally, the probe-set argument "Chromium_Mouse_Transcriptome_Probe_Set_v1.0.1_mm10-2020-A.csv" and the chemistry argument "MFRP" were applied. The resulting gene expression matrices were processed individually in R (v.4.4.0) using Seurat (v.5.0). Filters were applied to retain cells with more than 400 genes, more than 300 counts, and less than 10% mitochondrial reads. DoubletFinder was used to identify and remove doublets, with an expected doublet rate of 6.5–7.3% based on the loading rate.

Data normalization, scaling, and dimensionality reduction via principal component analysis (PCA) and clustering were performed to define distinct cell populations. Visualization using Unified Manifold Approximation and Projection (UMAP) aided interpretation. Marker genes for each cluster were identified using the Seurat function FindAllMarkers to define cell types or sub-cell types. Differential expression analysis across conditions and clusters was conducted using the FindMarkers function, employing the Wilcoxon rank-sum test. Gene annotation was performed by using STRING (https://string-db.org/). Of note, we found that one mouse sample contained a small number of cells annotated as neurons, while all other samples had zero neuron cells. This suggests that the neuron annotation was likely an isolated artifact rather than a systematic misclassification. We also observed that activated astrocytes were annotated across all mouse samples, but the cell counts were minimal, again suggesting an isolated artifact. To avoid confusion and ensure clarity in the data presentation, we have excluded both neuron and activated astrocyte annotations from the UMAP visualization.

### Statistical analysis

Statistical significance was determined as appropriate and as stated in the figure legends. The p value for RT-PCR results was calculated using the ΔCt values from different experimental groups. Significance was considered as p < 0.05.

## Results

### No significant age-related differences were observed in weight loss, lung-to-body weight ratio, or total BAL immune cell counts up to 7 days post-IAV infection

**Fig. 1:**
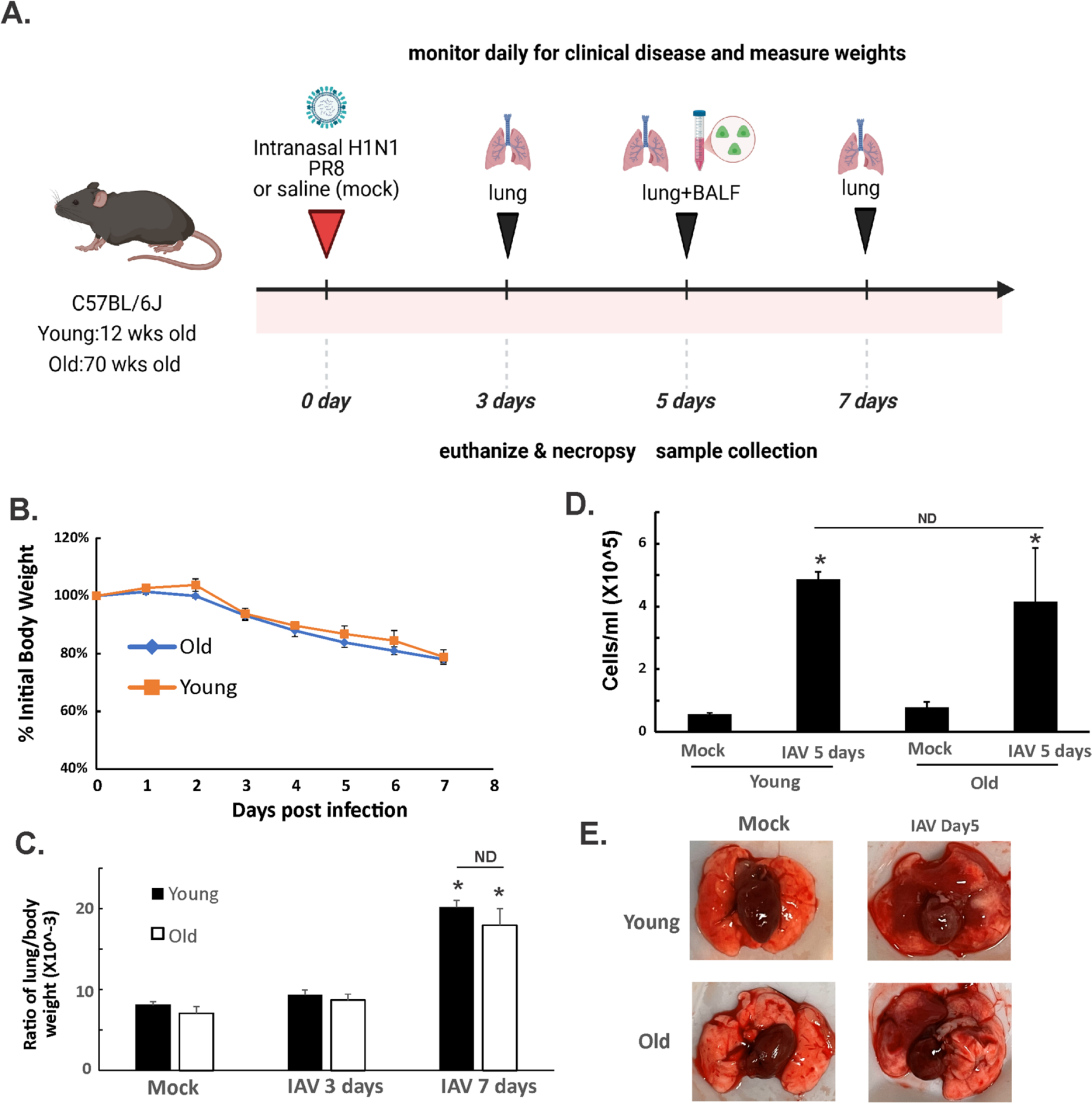
Weight loss, lung-to-body weight ratio and lung cellularity after IAV infection in young and old mice. Mice were intranasally inoculated with IAV PR8 at 200 PFU/mouse. Mock-treated mice were inoculated with viral diluent (PBS). (A) Schematic of the experimental plan on IAV infection to old and young mice. The image was created with BioRender.com. Body weights (B) were monitored daily. Body weight data were normalized to each mouse’s starting body weight. Lung tissue was harvested at day 3 and 7 after infection and ratio of lung-to-body weight (C) was determined. Data are expressed as mean ± standard deviation (n ≥ 8 for all groups; total mice = 18 in this experiment). Bronchoalveolar lavage fluid (BALF) was harvested at day 5 after infection. Total immune cells (D) in BALF were determined. Data are expressed as mean ± standard deviation (n ≥ 4 for all groups; total mice = 18 in this experiment). ND = no significant difference between the two groups. (E) Gross pathologic images of IAV-infected lungs. The image shown is representative of 5 mouse lungs from each treatment group. * denotes significant difference between the IAV infected and mock groups, p < 0.05. Data are expressed as means ± SEM

Young (12-week) and old (70-week) C57BL/6 J mice were infected intranasally (i.n.) with IAV PR8 strain. The mock group was inoculated with an equal volume of PBS as a negative control (Fig. 1A). The primary aim of this study was to investigate age-related changes in innate immunity in old mice. The innate immune response to influenza is characterized by a rapid onset, typically occurring within the first few days post-infection. Specifically, the initial inflammatory wave emerges between days 1–3, followed by a secondary wave between days 3–7 [24]. Thus, we selected 3, 5 and 7 dpi to sacrifice the animals and collect the samples. The percentage of weight loss was similar in all infected young and old mice and the weights continued to decrease up to 7 days post-infection (dpi), which was the experimental end point (Fig. 1B). IAV-induced lung injury was characterized by examining the lung-to-body weight ratio on 3 and 7 dpi, an indicator of acute lung injury (ALI) [25, 26]. IAV infection did not cause a significant change in the lung-to-body weight ratio in either age group at 3 dpi. However, the lung-to-body weight ratios were significantly increased at 7 dpi in both young and old mice as compared with mock-infected animals although there were no differences between the young and old mice (Fig. 1C).

We determined the total inflammatory cell numbers in bronchoalveolar lavage fluids (BALF) at 5 dpi and found IAV infection increased total viable leukocyte counts in BALF. Again, there was no significant difference in total inflammatory cell numbers between young and old mice (Fig. 1D). Gross appearance also demonstrated similar severity of lung hemorrhage in young and old mice (Fig. 1E).

### Aged mice had significantly higher viral loads and lower type I IFN in the lungs at 3 days after IAV infection

**Fig. 2:**
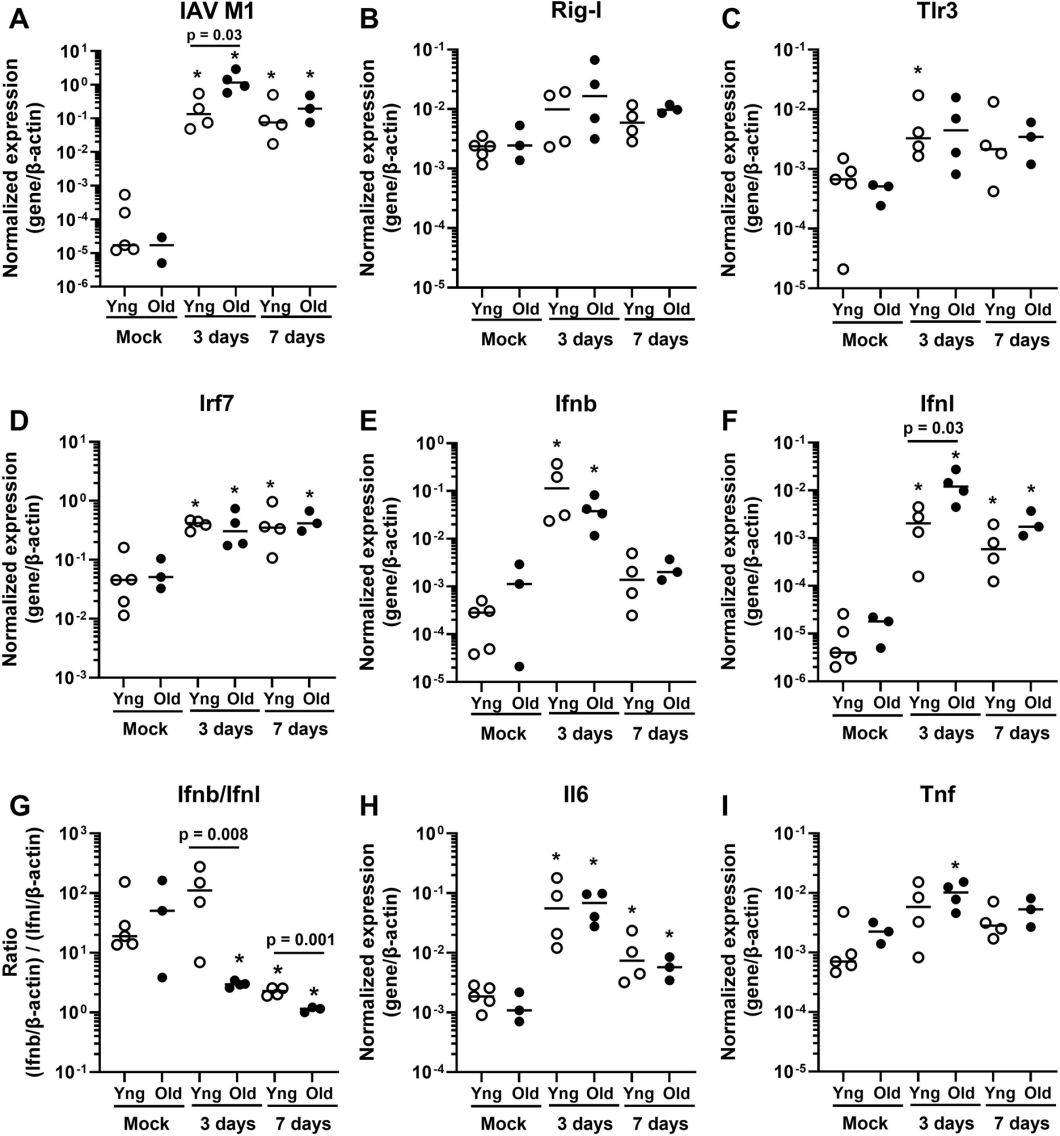
IAV replication is greater, and infection elicits lower *Ifnb* and greater *Ifnl* expression in the lungs of old mice compared with young mice. The mice were intranasally inoculated with IAV at 200 PFU/mouse. Mock-treated mice were inoculated with PBS. The mice were sacrificed at day 3 and 7 post infection and mRNA levels in the lungs were assessed by qRT-PCR and normalized by β-actin. Statistical analysis of gene expression was performed by the Kruskal–Wallis test with a Benjamini, Krieger and Yekutieli post-test analysis with FDR set at q < 0.05. Results in panels A and F were first transformed (log_2_) then analyzed by one-way ANOVA with Dunnett's post-hoc test. Significant results are represented by a (*). Comparisons between age groups at the same time point are analyzed by Mann–Whitney test with a level of significance set at p < 0.05. Significant p values are shown (n ≥ 3 for all groups; total mice = 23 in this experiment)

Next, we assessed the magnitude of IAV replication by measuring mRNA expression of the IAV M1 gene by qRT-PCR (Fig. 2A). We also measured mRNA expression of pattern recognition receptors (PRRs) and their downstream cytokines during infection in mouse lung (Fig. 2 B-I). Old mice were less able to control lung viral replication at 3 dpi with significantly greater viral M1 mRNA expression than that seen in young mice (Fig. 2A). At 7 dpi, differences between the age groups did not reach statistical significance. Even though the mock mice are not infected with IAV, there is a weak signal in the qRT-PCR assay due to non-specific amplification at higher cycle numbers that is 4 orders of magnitude lower in the mock-treated mice than IAV-infected mice and is considered biologically irrelevant (Fig. 2A). Both RIG-I and TLR3 PRR mRNA levels appeared to be higher in IAV-infected mice on day 3 after infection, although only the increase in TLR3 reached statistical significance (Fig. 2B, C). mRNA levels of the transcription factor IRF7 were also significantly induced by IAV infection (Fig. 2D). Notably, there was no statistical difference in the mRNA levels of these genes between IAV-infected young and old mice.

We then measured IFN-β (*Ifnb*) and IFN-λ (*Ifnl*) mRNA expression levels to assess type I and III IFN induction by IAV. Analysis showed there were significant increases in both *Ifnb* and *Ifnl* at 3 dpi in both young and old mice (Fig. 2E, F). However, the magnitude of *Ifnb* expression in young mice appeared greater (fivefold) than that in old mice, although this difference did not reach statistical significance (Fig. 2E). Old mice produced significantly more *Ifnl* mRNA than young mice (Fig. 2F) at day 3. This also appeared to occur at day 7 although this difference did not reach statistical significance at this time point. The results suggest that old mice had diminished upregulation of *Ifnb* during IAV infection but produced greater amounts of *Ifnl* mRNA than the younger cohort. The imbalance of *Ifnb* and *Ifnl* expression was more apparent when *Ifnb*/*Ifnl* ratios were plotted (Fig. 2G). At both day 3 and day 7, *Ifnb*/*Ifnl* ratios were significantly greater in the young mice. This result supports our central hypothesis that the balance of early IFN responses to IAV infection is disrupted in aged mice, with specific impairment in type I IFN induction in the lungs.

Additionally, the ability to upregulate IFNs over their baseline production in response to IAV infection was markedly different between the age groups. At 3 dpi, *Ifnb* increased in young mice by 659 ± 695 (mean ± SD) fold over mock compared to only a 31 ± 22-fold increase in old mice (p = 0.015, Unpaired, 2-tailed t-test of log_2_-transformed values). Conversely, *Ifnl* increased over baseline to a greater degree in old mice (922 ± 644-fold increase) than in young mice (241 ± 206-fold increase) but without a statistically significant difference (p = 0.09). By 7 dpi., the fold changes in both age groups for both Type I and Type III IFN had declined and were not significantly different between the ages.

At 3 dpi, robust, but similar IL-6 (*Il6*) induction by IAV was observed in both young and old mice (Fig. 2H). By 7 dpi, *Il6* levels remained elevated in both groups. *Tnf* expression was also increased at 3 dpi in both groups but did not achieve statistical significance in young mice due to high variance (Fig. 2I).

**Fig. 3:**
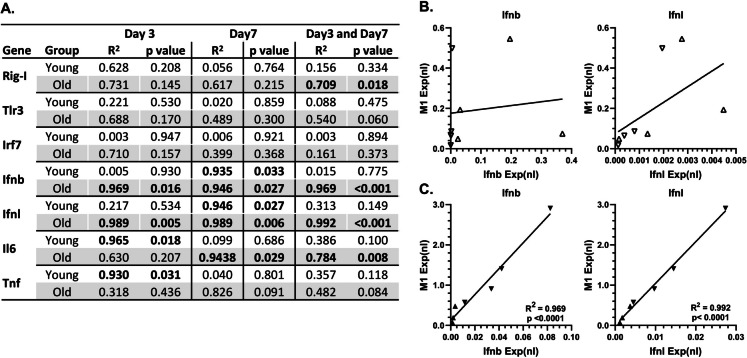
Magnitude of IAV pulmonary infection correlates positively with expression of pro-inflammatory mediators in the lungs. Young and old mice were infected intranasally with 200 PFU/mouse IAV as described above. Lung gene expression was measured by qRT-PCR and normalized to β-actin. (A) Correlation (R^2^) of IAV M1 gene and host gene expression and p values were determined by simple linear regression. Significant values are bolded. (B, C) Scatter plots show correlations between M1 gene expression and *Ifnb* (left panel) and *Ifnl* (right panel) in young (B) and Old (C) mice. Symbols represent individual young (∇,∆) and old (▼,▲) mice harvested 3 (∇, ▼) and 7 (∆,▲) days after pulmonary infection with IAV. Gene expression in the lungs was determined by qPCR and normalized to β-actin. Significant correlations and p values are shown (n ≥ 3 for all groups; total mice = 23 in this experiment)

Analyzing expression of host defense genes in the context of lung viral load (M1 expression) showed significant correlations at 3 dpi between viral load and expression of *Il6* and *Tnf* in young mice, and *Ifnb* and *Ifnl* in old mice (Fig. 3A). By 7 dpi, the correlation between *Il6*/*Tnf* induction and viral loads in young mice was lost; instead, viral loads correlated with *Ifnb* and *Ifnl*. In contrast, in old mice, not only did the correlation between viral loads, *Ifnb* and *Ifnl* expression persist, but *Il6* mRNA levels also correlated with viral loads. (Fig. 3A). When the analysis was expanded to measure correlation at all time points for both ages of mice, although there were no significant correlations in young mice including for *Ifnb* and *Ifnl* (Fig. 3A, B), in old mice M1 expression was significantly correlated with levels of Rig-I, *Ifnb*, *Ifnl*, and *Il6* (Fig. 3A, C). These data show that, although overall the magnitude of IAV infection is positively correlated with host PRR and cytokine responses that contribute to anti-viral activity, there is an important difference between the responses in old and young mice.

### Aged mice had enhanced late lung tissue injury after IAV infection

**Fig. 4:**
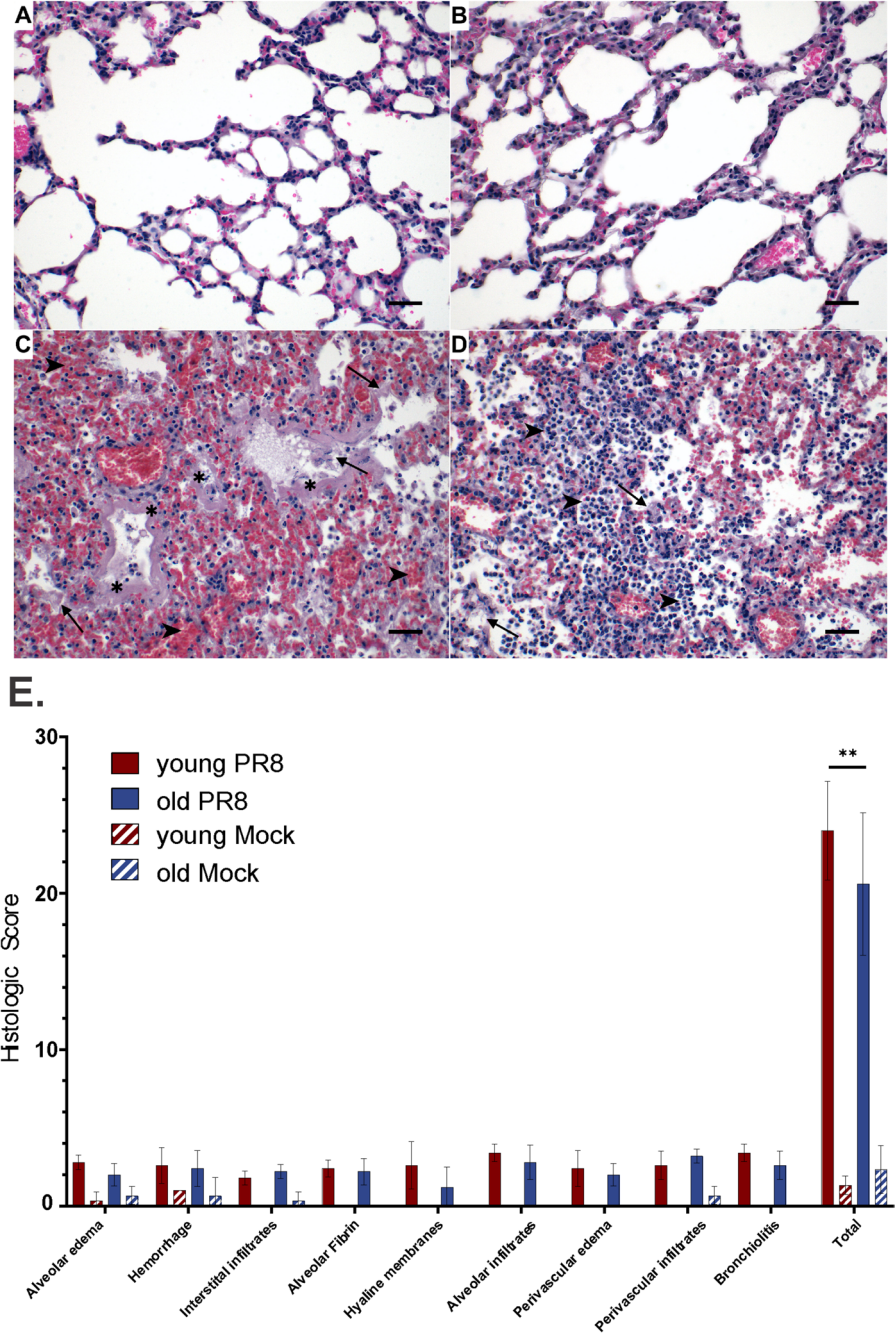
Mouse lung tissue pathological changes at 5 dpi. Mice were intranasally inoculated with IAV at 200 PFU/mouse. Mock-treated mice were inoculated with PBS. Lung tissue was harvested at day 5 after infection. Compared to uninfected mice (A), IAV-infected young (C) mouse lungs exhibited a significant degree of pulmonary pathologic changes with diffuse alveolar damage, prominent hyaline membranes (asterisks), alveolar fibrin (arrows), and hemorrhage (arrows). Increased lung pathology was also observed in IAV-infected old mice (D) compared to uninfected old mice (B), characterized by alveolar fibrin (arrows) and marked inflammatory infiltrates (arrowheads). Histopathologic evaluation and scoring of IAV infection were determined by a pathologist blinded to treatment group (E). Scale bar = 50 µM (20X). Statistical analysis of histopathologic scores for specific individual specific changes and the overall compiled score was performed by two-way ANOVA with Turkey test. ** denotes significant difference between the two groups, p < 0.01 (n ≥ 3 for all groups; total mice = 23 in this experiment)

Lung tissues were evaluated for histopathological changes. Figure 4 A and B were uninfected normal lungs from young and old mice. At 5 dpi, IAV-infected young mice exhibited characteristic features of virus-induced, diffuse alveolar damage (DAD) such as marked alveolar edema, fibrin exudation, hemorrhage, and prominent hyaline membrane formation (Fig. 4C). IAV-infected old mice exhibited similar histological lesions, although the degree of hyaline membrane formation was significantly greater in young mice compared to old mice (p = 0.0195; (Fig. 4D). Total lung pathology scores were also significantly higher in young mice compared to old mice at this early time point (p = 0.0036; Fig. 4E). Lung histopathology of mock-infected control mice (young mock and old mock) was unremarkable and within normal limits. The data suggested that the old mice had suppressed innate immune responses at the early stage of IAV infection.

**Fig. 5:**
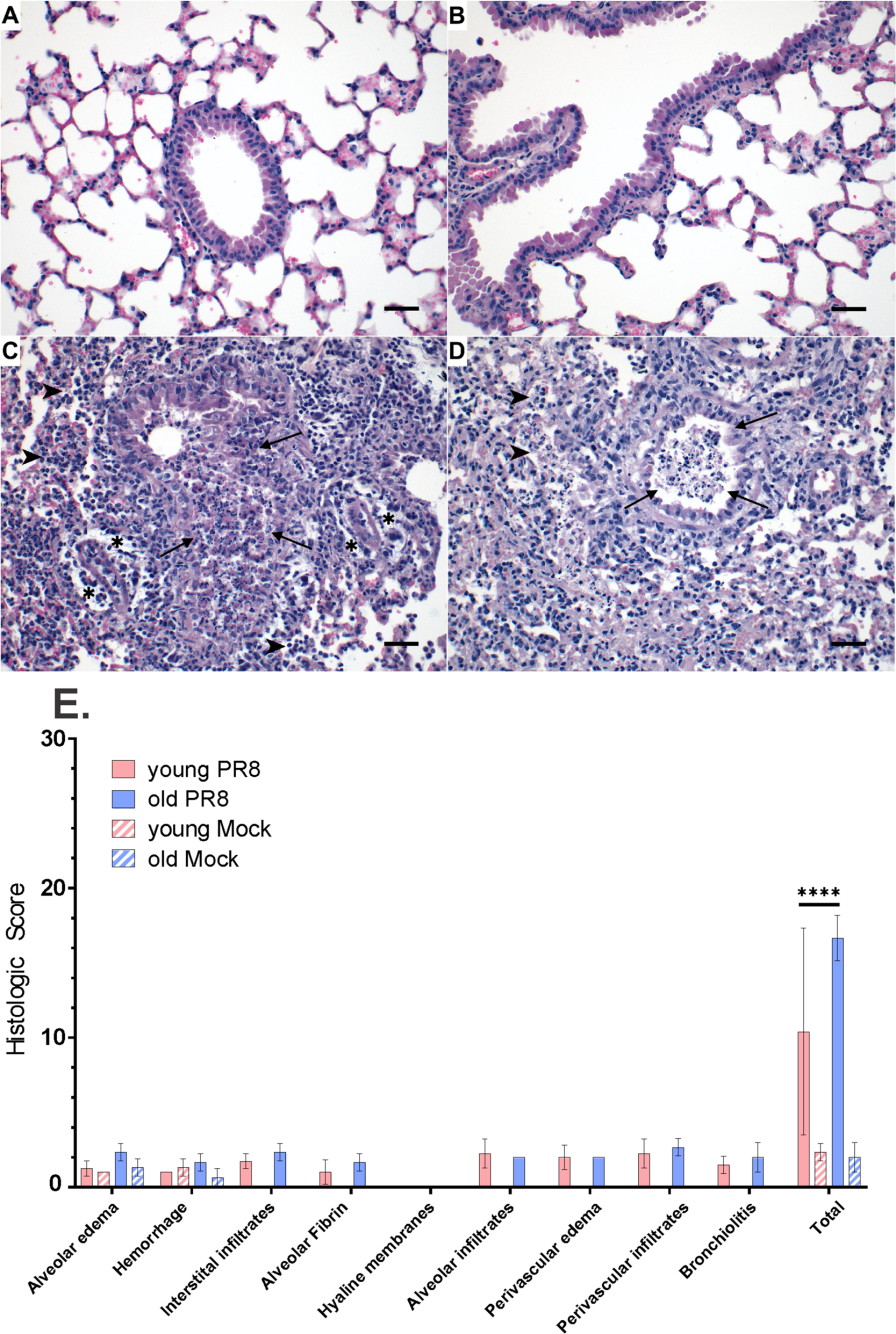
Mouse lung tissue pathologic changes at 7 dpi. Mice were intranasally inoculated with IAV at 200 PFU/mouse. Mock-treated mice were inoculated with PBS. Lung tissue was harvested at 7 dpi. Compared to uninfected young mice (A), young mice with IAV infection (C) exhibited significantly enhanced pulmonary pathologic changes characterized predominantly by increased alveolar infiltrates (arrowheads), perivascular mononuclear infiltrates and edema (asterisks), and bronchiolitis (arrows). Increased lung pathology – such as bronchiolitis (arrows) and alveolar infiltrates (arrowheads) – was also increased in old mice infected with IAV (D) compared to uninfected old mice (B), and IAV-infected old mice exhibited a slightly greater degree of alveolar edema, hemorrhage, and alveolar fibrin compared to young mice with IAV infection (C). Histopathologic evaluation and scoring of IAV infection were determined by a pathologist blinded to treatment group **(E)**. Scale bar = 50 µM (20X). Statistical analysis of histopathologic scores for specific individual specific changes and the overall compiled score was performed by two-way ANOVA with Turkey test. **** denotes significant difference between the two groups, p < 0.0001(n ≥ 3 for all groups; total mice = 23 in this experiment)

For mouse lung at 7 dpi, all young mice infected with IAV exhibited a significant, but variable degree of pulmonary pathology that was primarily characterized by marked alveolar infiltrates, perivascular mononuclear infiltrates and edema, and bronchiolitis (Fig. 5C). Histological lesions were more uniform in old mice infected with IAV at 7 dpi (Fig. 5D), with marked bronchiolitis and increased alveolar infiltrates predominating. In contrast to the results at 5 dpi, at this later stage of viral infection, total lung pathology was significantly increased in old mice when compared to young IAV-infected mice (p < 0.0001; Fig. 5E). For young mice, the total scores dropped 13.6 (P < 0.0001) from day 5 to day 7. For old mice, the total scores only dropped 3.9 (P = 0.0035) from day 5 to day 7. It seems that, despite the more robust initial *Ifnb* and inflammatory response in young mice, there were already signs of tissue healing/resolution of inflammation, the healing/resolution phase might take longer in old mice.

### Old mice had greater inflammation-related gene expression in lung innate immune cells at 7 dpi

**Fig. 6:**
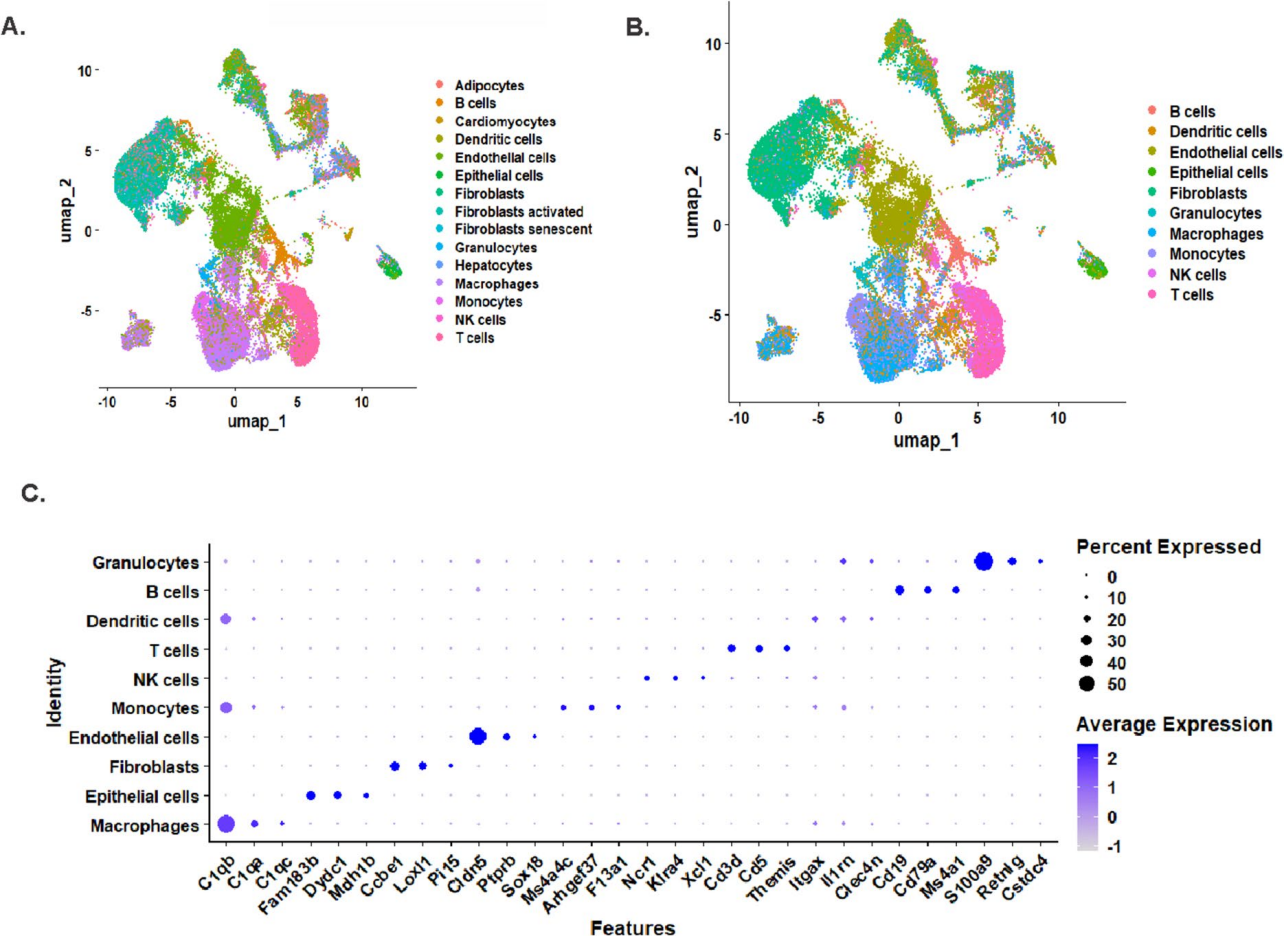
scRNA-seq landscapes of mouse lungs Mice were intranasally inoculated with IAV at 200 PFU/mouse. Lung tissue was harvested at 7 dpi. (A) Uniform manifold approximation projection (UMAP) visualization of single-cell RNA sequencing data from mouse lungs. Each point is an individual cell colored by cell type. (B) UMAP showing 11 cell populations which were further selected as possible innate immune related cells. (C) Dot plot showing key markers used to identify cluster identities. Color intensity denotes expression level; dot size denotes percentage of cells in each cluster expressing a given gene. UMAP axes represent dimensionally reduced components without standard units

Next, we applied single-cell RNA sequencing (scRNA-seq) analyses to our mouse model and compared the lung immune responses of old and young animals at cellular & molecular levels. We focused particularly on the lungs at 7 dpi, a critical time point when differences in histopathological changes between the old and young animals begin to emerge. Seven days after IAV infection, mice were euthanized, and their lungs were perfused and collected. A total of 29,402 cells (young mock 2,844 cells, young PR8 6,256 cells, old mock 2,753 cells, and old PR8 11,305 cells) with 28 distinct clusters were revealed by this analysis. These clusters were further characterized into 17 cell populations (Fig. 6A) using FindAllMarkers. Out of the 17, 11 cell populations were further selected as they have a role in innate immune responses (Fig. 6B) including natural killer (NK) cells, macrophages, monocytes, granulocytes (including neutrophils, eosinophils and basophils), dendritic cells (DC), B cells, T cells, endothelial cells, and epithelial cells. Figure 6C shows a dot plot of the mean expression levels of canonical marker genes for these 11 lung cell populations.

**Fig. 7:**
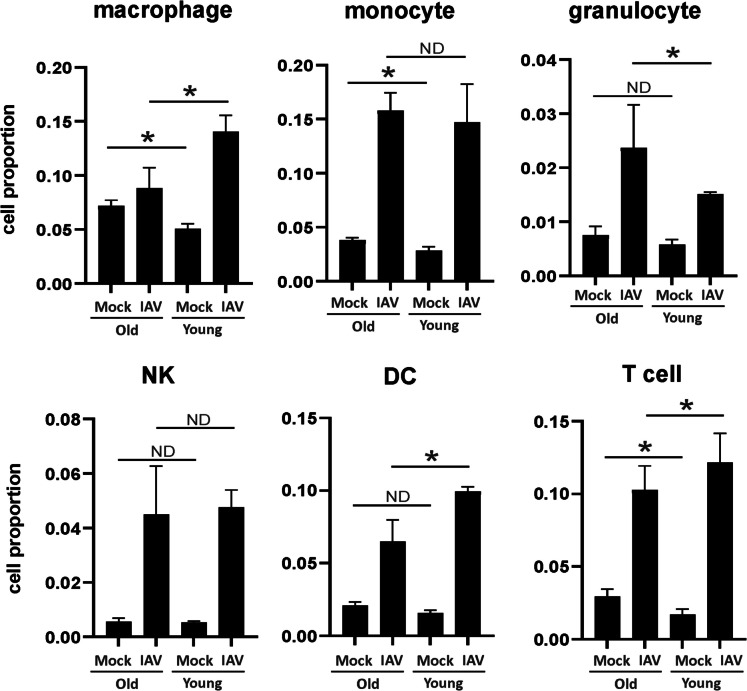
Cell frequencies as a fraction of total lung cells over time separated by age and infection groups. Mice were intranasally inoculated with IAV at 200 PFU/mouse. Mock-treated mice were inoculated with PBS. Lungs were harvested at 7 dpi. Columns represent the mean for each group. P-values are calculated in a two-way ANOVA with a Tukey test for multiple comparisons (n = 2 for mock mice, n = 3 for IAV-infected mice; total mice = 10 in this experiment)

We compared the relative distributions of immune cell compartments between the mock and IAV PR8 infection groups, as well as between the old PR8 and young PR8 infection groups. In IAV-infected mice, monocytes and macrophages constituted the largest proportion of the 11 immune cell clusters, making up 47.4% of these clusters in old mice and 47.9% of these clusters in young mice. In uninfected animals, old mice had significantly higher numbers of macrophages (7.2% of total cells) than did young mice (5.1% of total cells; Fig. 7). However, following IAV infection, young mice had significantly higher macrophage counts (14.1% in total cells) than did old mice (8.8% in total cells). Regarding monocytes, both old and young infected mice showed similar increases upon IAV infection. This suggests that despite having a higher baseline number of macrophages, old mice have an impaired ability to augment this number through increased recruitment over time or by differentiation of recruited monocytes into macrophages.

There was significant recruitment of granulocytes to the lungs in both young and old mice during IAV infection (Fig. 7). IAV-infected aged mice exhibited significantly higher numbers of granulocytes compared to infected young mice. These data suggested that granulocytes are retained in the lungs of aged mice later during IAV infection. Persistent excessive granulocyte activity at 7 dpi in old mice could lead to impaired lung function by causing airway hyperresponsiveness, mucus production, and tissue damage, consistent with our histologic findings (Fig. 5). As for NK and DC cells, both were significantly increased after infection (Fig. 7). However, there was no difference in NK numbers between old and young mice. In contrast, after infection young mice exhibited significantly higher numbers of lung DCs compared to old mice. Importantly, young mice exhibited significantly higher numbers of T-cells at 7 dpi compared to old mice. Together, this suggests that young mice also display more robust systemic recruitment of T cells to the lungs than the old mice.

**Fig. 8:**
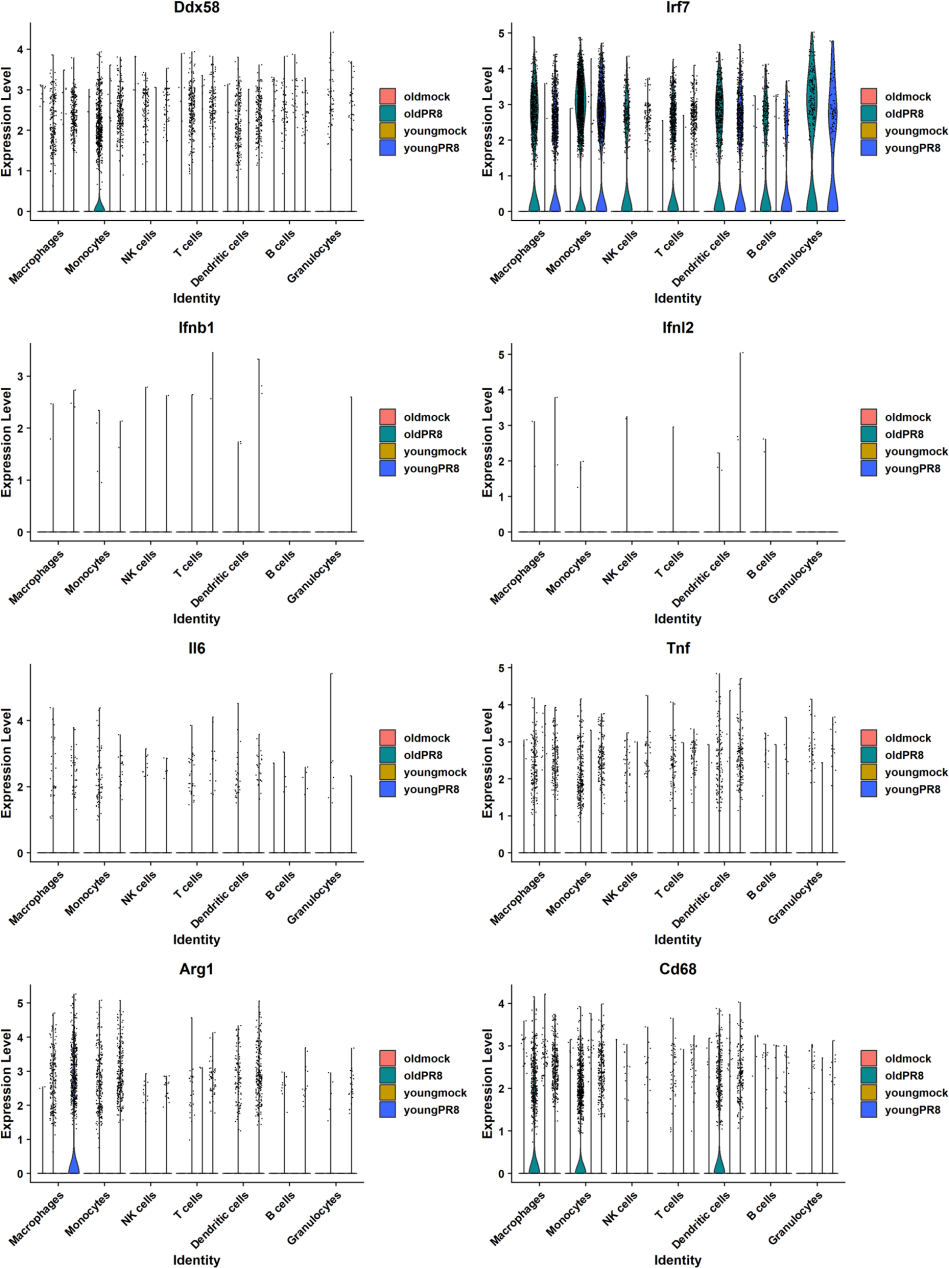
Old mice had more inflammation-related gene expression in lung innate immune cells at 7 dpi. Mice were intranasally inoculated with IAV at 200 PFU/mouse. Mock-treated mice were inoculated with PBS. Lungs were harvested at 7 dpi. Violin plots illustrating the probability distributions of gene expression across lung innate immune cell populations. The cell populations were compared. The plots represent gene expression counts (n = 2 for mock mice, n = 3 for IAV-infected mice; total mice = 10 in this experiment)

We then created violin plots to analyze the probability distribution of gene expression across various clusters. At 7 dpi, old mice exhibited higher expression levels of *Ddx58* (RIG-I), *Irf7*, *Il-6*, and *Tnf* in macrophages, monocytes, NK cells, DC, and granulocytes compared to young mice (Fig. 8). Current data suggest macrophages do not follow a rigid classification of polarization and can exist in a number of states [27]. Thus, we have opted to categorize macrophages as M1-like (pro-inflammatory) and M2-like (anti-inflammatory, involved in tissue repair and immune regulation) in this report. We evaluated markers of M1/M2-like macrophage polarization in the lungs. *Arg1* is a well-known marker of M2-like macrophages, which possess anti-inflammatory properties that maintain tissue homeostasis and resolve inflammation [28]. CD68 molecule is used as pan-marker of macrophages and the co-expression of CD68 and HLA-DR indicates the M1-like phenotype of the macrophages [29]. In our study, we utilized multiple well-established markers to characterize macrophage polarization, including CD68 and HLA-DR for phenotypic identification and Nos2/Arg1 for functional classification. While CD68 and HLA-DR co-expression is commonly used in immunohistochemical studies to identify M1-like macrophages, Nos2 and Arg1 are widely accepted transcriptomic markers for M1 and M2 polarization, respectively, especially in scRNA-seq analyses. Notably, old mice expressed significantly less *Arg1* and more CD68 than young mice, indicating a predominance of M1-like macrophages in the older group.

### Identification of unique highly expressed genes in macrophages in IAV-infected old mice

**Fig. 9:**
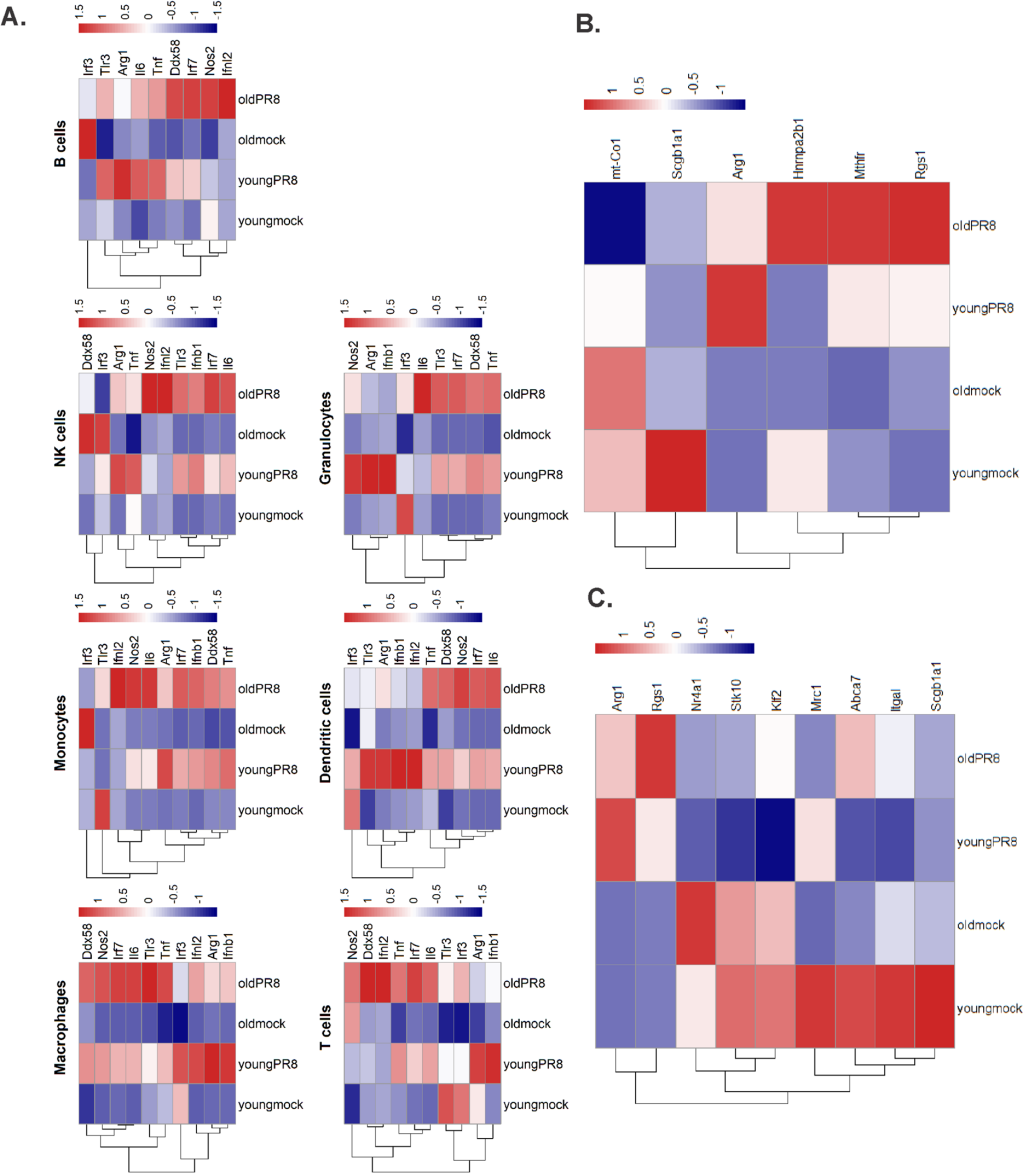
Identification of differentially expressed genes (DEGs) in macrophages and monocytes in IAV-infected old mice at 7 dpi. Mice were intranasally inoculated with IAV at 200 PFU/mouse. Mock-treated mice were inoculated with PBS. Lungs were harvested at 7 dpi. (A) Heatmaps showing DEGs in innate immune cells in mouse lungs. Significant DEGs in lung macrophages (B) and monocytes (C) are shown between young and old mice during IAV infection (n = 2 for mock mice, n = 3 for IAV-infected mice; total mice = 10 in this experiment)

We generated heatmaps to visualize the transcriptional signatures of inflammation-associated genes in each type of immune cells (Fig. 9A). Generally, IAV infection induced the expression of *Ddx58*, *Irf7*, *Il-6*, *Tnf* and *Nos2* in both young and old mice at day 7 dpi. However, old PR8-infected mice showed higher expression levels of *Ddx58*, *Irf7*, *Il-6*, and *Tnf* in macrophages, monocytes, NK cells, DCs, and granulocytes compared to young PR8-infected mice at this time point. In the context of lung inflammation, *Arg1* and *Nos2* serve as key markers distinguishing M2- and M1-like macrophage phenotypes, respectively. High *Arg1* expression indicates M2-like macrophages, which promote tissue repair, while high *Nos2* expression signifies M1-like macrophages, which contribute to inflammation within the lung environment. In both macrophages and monocytes, PR8-infected old mice expressed significantly more *Nos2* and less *Arg1* than young mice. These data demonstrate that proinflammatory cytokine-related genes were highly upregulated following IAV infection, with aging significantly sustaining this upregulation at 7 dpi.

Given the role of macrophages and monocytes as key mediators of inflammatory responses in the lung, we focused on identifying differentially expressed genes (DEGs) between young and old groups during IAV infection. Figure 9 highlights all DEGs with an FDR-adjusted p-value of less than 0.05 and a fold change greater than 2. Notably, three genes were significantly upregulated in macrophages from old mice compared to their young mouse counterparts: *Hnrnpa2b1* (Heterogeneous nuclear ribonucleoprotein A2B1), *Mthfr* (methylenetetrahydrofolate reductase), and *Rgs1* (Regulator of G Protein Signaling 1). *Hnrnpa2b1* plays a crucial role in initiating IFN-alpha/beta production and enhancing cytoplasmic antiviral signaling [30]. The *Mthfr* gene encodes an enzyme essential for folate metabolism. Variants in the *Mthfr* gene are associated with an increased risk of cognitive decline, including memory loss, confusion, and depression [31]. *Rgs1* encodes a member of the regulator of G-protein signaling family, which is involved in transmitting signals from hormones to neurotransmitters via G proteins and G protein-coupled receptors (GPCRs). Interestingly, in both macrophages and monocytes from young mock-infected mice these cells had significantly higher expression of *Scgb1a1* (Secretoglobin Family 1 A Member 1), which possesses anti-inflammatory and immunomodulatory properties. This protein binds to small hydrophobic molecules, such as prostaglandins and phospholipids, to mitigate inflammation. Overexpression of *Scgb1a1* in the airways can reduce ventilator-induced lung inflammation and injury, whereas a deficiency in Scgb1a1 can lead to early activation of inflammatory pathways in alveolar macrophages [32].

## Discussion

Despite extensive research on IAV infection, a mechanistic understanding of the host response and host–pathogen interactions remains quite limited, particularly with regard to the effects of aging on the critical immune responses to IAV. The effects of IFNs on inflammatory responses and lung injury in age-associated changes are also poorly understood.

We found that old mice had a diminished capacity to control viral replication in the lung, as reflected by relatively higher viral M1 gene expression in old animals. This is most likely due to the reduction of the IFN-β and subsequent ISG response in these animals. Consistent with this hypothesis, Pillai et al. showed that, although monocytes and macrophages from older human adults have intact RIG-I signaling and activation of proinflammatory cytokines, these cells have defective signaling related to production of type I IFNs [33]. Using human lung tissue from donors aged 22–68 years, Nguyen et al. found that while IAV infection induces a rapid IFN response, this response wanes with age. Specifically, IAV-infected lung tissue resident memory CD8 + T cells from aged donors produced less type I IFN, when compared with the same cells from young donors [34]. Historically, viruses that have evolved to develop mechanisms of disrupting activation of type I IFN are likely to be highly pathogenic. One notable example is the 1918 pandemic IAV strain [35, 36]. Our findings demonstrating delayed viral clearance in aged animals is consistent with prior results by other investigators. One mouse model showed that IAV infection generated a prolonged viral load plateau in aged animals, with delayed virus clearance at 11 dpi instead of 9 dpi seen in young mice [37]. In a primate model, increased viral loads were shown to correlate with increased disease severity in aged macaques infected with the 2009 pandemic IAV [38]. Therefore, it is likely that aging-related impairments in the early robust type I IFN response to IAV diminish the host’s ability to contain viral replication and exacerbate the infection. Kasmani et al. showed that high-resolution atlas provides a comprehensive analysis of age-related changes in the lung’s response to influenza infection, particularly through the integration of immune, epithelial, and stromal compartments using scRNA-seq, cytokine profiling, and detailed cell-type annotation [39]. While their study used older mice (18–19 months, about 2.5–3 months older than we did) and sampled across multiple infection stages, our work complements theirs by focusing on early innate responses in 16-month-old mice, thereby contributing additional insight into the age spectrum of host–pathogen interactions. Our study employed a markedly different infective dose compared to theirs, 200 PFU versus 50 PFU per mouse. This variation in inoculum is highly relevant and provides important additional insight, especially considering that human exposure to viral load can vary widely in real-world scenarios.

The host cytokine response shapes and diversifies the arsenal of independent immune effector mechanisms. Thus, any pathogens that overcome the direct antiviral effects of type I IFNs will have to contend with other antiviral activities that may attempt to compensate for viral-mediated inhibition of the normal host protective response [40]. This may explain why we found increased type III IFN in aged mice. On the other hand, this finding may be related to the higher viral load seen in these animals as IFN-λ levels generally depend on input viral dose. In our experiments, there was a tight correlation between M1 and IFN-λ expression, but this only occurred in the aged mice.

Type I and III IFNs are not only important in antiviral effects against IAV, but they are also involved in defining downstream innate and adaptive immune responses [16, 40]. Type III IFNs are critical regulators of neutrophil activation and DC development for optimal CD8 + T cell responses in mice [41–43]. However, higher type III IFNs disrupt tissue repair during pulmonary hyperinflammatory conditions and lead to late lung damage and triggering of excessive production of inflammatory cytokines [14]. Ahn et al. showed that a significant physiological consequence of type III IFN signaling is a reduction in epithelial barrier integrity, which not only makes it easier to recruit immune cells but also makes it possible for the bacterial pathogen *K. pneumoniae* to invade tissue [44]. Thus, excessive type III IFN may result in negative effects for the aged host to combat secondary bacterial infection [45].

The timing and magnitude of type I IFN are crucial to the outcome of a hosts’ response to viruses. We have previously shown that IFN-β administration to nonsmoking mice with normal IFN response led to uncontrolled inflammation, enhanced ALI, and decreased viral containment. However, early, but not late, IFN-β administration to cigarette smoke-exposed mice, which had suppressed innate responses, played a protective role against mortality and morbidity during IAV infection [46]. Aging, acting like cigarette smoke, may simply impair the capacity to develop an optimal type I IFN response early after infection, which is crucial to reducing pathology through both controlling viral replication and regulating the immune response to diminish immunopathology [47]. An early and effective, i.e. normal, type I IFN response during viral infection protects against severe inflammation, but delayed and dysregulated type I IFN production can increase disease symptoms and worsen outcomes. Hence, it is likely that aging-related delays or deficits in the ability to promptly produce type I IFNs underpin worsened illness after infection.

We observed that IAV-infected young and old mice exhibited comparable weight loss, lung-to-body weight ratios, and total inflammatory cell numbers in the BALF (Fig. 1). However, since lung samples were only collected up to 7 dpi as snapshots, it is possible that measures of lung inflammation may show significant increases in old mice at later time points. This study serves as an initial investigation into the effects of aging on lung gene expression and innate immune responses to IAV infection. Our focus was on the first 7 dpi, utilizing the smallest sample size necessary to ensure adequate statistical power while minimizing animal usage in accordance with IACUC guidelines and resource conservation. Moving forward, we will expand our research to assess infections at later stages in future studies.

Using single-cell RNA sequencing of mouse lungs, we observed significant differences in inflammatory innate responses to IAV infection between old and young animals in specific lung populations. At 7 dpi, old mice exhibited sustained local inflammatory responses, with higher expression levels of *Ddx8*, *Irf7*, *Il6*, and *Tnf* across various immune cells, including macrophages, monocytes, NK cells, DCs, and granulocytes, compared to young mice. DEG analysis found that *Rgs1* is significantly increased in macrophages in IAV-infected old mice. This gene appears to be important in monocyte–macrophage activation and recruitment, as it was upregulated in M1-polarized macrophages differentiated from healthy human peripheral blood mononuclear cells [48]. Our data suggest that lung macrophages in old mice are skewed towards an M1-like phenotype by 7 days after IAV infection. scRNA-seq also revealed that old mice had significantly lower Arg1 and higher Nos2 expression in macrophages and monocytes than did young mice. This low Arg1/Nos2 ratio is also consistent with a predominance of M1-like polarization of macrophages, suggesting ongoing acute inflammation in the lungs of old mice, while young mice had already shifted towards anti-inflammatory and tissue repair processes as evidenced by M2-like skewing at 7 dpi. Additionally, young mice showed significantly higher T-cell responses at 7 dpi compared to old mice. This implies that young mice not only regulated innate inflammation more appropriately but also demonstrated more efficient local activation or systemic recruitment of T cells than old mice.

DEGs are genes that exhibit significantly different expression levels between two or more groups. Identifying DEGs is an essential step in genetic research as it helps to understand the molecular pathways behind diseases or phenotypes. Additionally, DEGs can be used to find biomarkers for diagnosis or prognosis, as well as to create customized medications and therapies. For example, *Rgs1* is a member of the regulator of G-protein signaling (RGS) family. Leandro et al. found that increased expression of *Rgs1* in the PBMCs of Alzheimer's disease (AD) patients. So *Rgs1* may serve as a peripheral biomarker for AD patients [49, 50]. Our previous publication showed that the brains of old animals have unique gene and pathway changes in responses to IAV infection that might cause neuro-cognitive dysfunction after acute infection [51]. The elevated *Rgs1* induction in lung macrophages may serve as a biomarker for IAV-induced activation of the peripheral innate immune system triggering neuro-cognitive pathologies in the brain. Additionally, *Rgs1* acts as a negative regulator, inhibiting the chemotaxis of cytotoxic T lymphocytes and Th1 cells toward tumor-associated chemokines [52]. Animal experiments have shown that Rgs1 inhibits chemotaxis of macrophages, causing them to accumulate at sites of inflammation during chronic inflammation as they are less sensitive to signals that cause these cells to migrate out from these sites [48]. Thus, it is suggested that *Rgs1* causes macrophages to accumulate locally by desensitizing chemokine receptors, resulting in prolonged inflammation. Our study identified *Rgs1* as a potential therapeutic target for aged individuals infected with IAV. Since the binding of *Rgs1* to G proteins accelerates the termination of cellular signaling and exacerbates disease by causing macrophages to aggregate, *Rgs1* inhibitors may competitively bind to *Rgs1*, potentially modulate macrophage function and ameliorate prolonged inflammation, which will promote better outcomes in diseases featuring extensive inflammation, such as viral infection.

We recognize our study has some limitations. In our study, we used 70-week-old (approximately 16-month-old) C57BL/6 J mice as a model for aging in humans. While Jackson Laboratories defines 18-month-old mice as “old,” age equivalency between mice and humans can vary depending on the parameters used. Some sources suggest that 70-week-old mice correspond to roughly 60-year-old humans [53, 54]. Testing mice older than 70 weeks could provide additional insights, but it also presents challenges. Older mice are more susceptible to IAV and other infections, which could necessitate increasing the number of animals or reducing the inoculum to avoid excessive mortality. These adjustments may introduce survivor bias into the study [55, 56]. Importantly, prior research using this model has shown that 70-week-old mice exhibit significant baseline differences in brain gene expression compared to younger (12-week-old) mice-differences that are consistent with aging [51]. Therefore, while similar trends might be observed in even older mice (e.g., 18–24 months), the magnitude of effects could be greater. Conducting such experiments would also likely result in substantial loss of animals due to infection-related mortality. Finally, as mortality in humans is significantly increased in the cohort aged 50–64, our data likely reflects age-related dysfunction that may cause increased mortality in this group. Another limitation of this study is the absence of protein-level validation to support transcriptomic findings. Although scRNA-seq provides high-resolution insights into gene expression dynamics, it does not directly confirm changes at the protein level. Future work will include complementary techniques such as flow cytometry, immunofluorescence, or immunohistochemistry to validate immune marker expression and enhance the functional relevance of our results.

## Conclusion

In conclusion, aging results in insufficient antiviral type I IFN responses early (day 3) after infection, as well as hyperinflammatory immunopathological processes late during IAV infection. The breakdown of a timely and adequately synchronized innate and adaptive immune response mediated by defective IFN responses is likely at least partially responsible for the age-associated rise in influenza severity. The ability to induce a potent, appropriate, and early IFN response may be helpful in aged people suffering from respiratory viral diseases.

## Data Availability

Single-cell RNA sequencing data from this paper are available in the GEO database with accession numbers GSE292515.
